# Comprehensive analysis of cuproptosis-related immune biomarker signature to enhance prognostic accuracy in gastric cancer

**DOI:** 10.18632/aging.204646

**Published:** 2023-04-07

**Authors:** Jie Li, Tian Yu, Juan Sun, Ziyang Zeng, Zhen Liu, Mingwei Ma, Zicheng Zheng, Yixuan He, Weiming Kang

**Affiliations:** 1 Department of General Surgery, Peking Union Medical College Hospital, Chinese Academy of Medical Sciences and Peking Union Medical College, Dongcheng, Beijing 100730, People’s Republic of China

**Keywords:** gastric cancer, cuproptosis, immune infiltration, prognostic signature, bioinformatics

## Abstract

Background: Gastric cancer (GC) is a malignant tumor with high prevalence and fatality. Cuproptosis is a recently identified copper-dependent programmed cell death mechanism. Multiple studies have demonstrated the profound impact of the immune microenvironment on tumor development. Hence, we decided to excavate the potential functional roles of cuproptosis-related immune genes (CRIGs) in GC and their values as biomarkers.

Methods: Cuproptosis- and immune-related genes were curated from top published studies on cell cuproptosis and cellular immunity. Transcriptome data and clinical information were obtained from TCGA, GTEx, and GEO databases. Cox and LASSO analyses were used to establish a prognostic signature for GC. Long-term prognosis, immune infiltration, immune checkpoint, and drug response were compared between signature groups. CRIG expression in GC scRNA-seq was analyzed. Immunohistochemistry was used to evaluate CRIG and cuproptosis regulator FDX1 in GC tissues.

Results: Seven CRIGs (ANOS1, CTLA4, ITGAV, CXCR4, NRP1, FABP3, and LGR6) were selected to establish a potent signature to forecast the long-term prognosis of patients. GC patients had worse prognosis and poor responses to chemotherapeutic drugs (5-Fluorouracil and paclitaxel) in the high-risk group. scRNA-seq revealed that CTLA4, ITGAV, CXCR4, and NRP1 enrichment in specific cell types regulated the progression of GC. Moreover, NRP1, CXCR4, LGR6, CTLA4, and FDX1 were elevated in GC tissues, with a positive correlation between their expression and FDX1.

Conclusions: To conclude, this study first provides insights into the functions of CRIGs in GC. Furthermore, a robust cuproptosis-related immune biomarker signature was established to forecast the long-term survival of GC patients accurately.

## INTRODUCTION

Gastric cancer (GC) is one of the most frequent malignant tumors worldwide, with its morbidity and cancer-related mortality ranking fifth and fourth worldwide, respectively [1]. New GC cases in China accounted for 44.1 % of the global new cases yearly. At the same time, GC-associated deaths accounted for more than 50 % of the total global deaths of GC. Such phenomena severely endangered people’s health, subjecting patients to significant economic and mental burdens [2]. Currently, tumor-node-metastasis (TNM) staging of postoperative specimens is a widely used clinical tool to forecast the long-term survival time of GC patients. However, studies have revealed that TNM staging still has significant limitations in clinical application [3]. Abundant studies have proved the efficiency of multi-gene signatures in distinguishing different cancer recurrence risks and improving the prognostic accuracy of patients [4–7]. Hence, it is essential to construct a potent signature for forecasting long-term prognosis and stratifying risk of GC patients to guide the precise treatment of individuals as a solution to a significant problem in GC.

Cell death, a critical biological regulation during the development of organisms, ensures the stability of the microenvironment by causing the death of damaged, aging, or excess cells [8]. Copper- and mitochondrial respiration-dependent cuproptosis is the most recently identified mechanism of programmed cell death, crucial for the malignant behavior of tumor cells and immune regulation in the tumor microenvironment (TME) [9–11]. The clinicopathological characteristics of the tumor and the immune response in the TME exert critical roles in determining the long-term prognosis of GC [12]. Immune evasion is one of the hallmarks of malignant tumors, aggravating it by inhibiting immune clearance [13]. Cytotoxic T-lymphocyte-associated protein 4 (CTLA4), a key immune checkpoint protein, is significantly upregulated in the T cells in tumors and represses the immune response upon binding to ligands CD80 or CD86 on antigen-presenting cells [14]. Chemokine receptor CXCR4, belonging to the G protein-coupled receptor (GPCR) family, impairs chemosensitivity and promotes cell migration and invasion, stemness, and tumorigenesis of GC [15–17]. In addition, leucine-rich repeat-containing GPCR 6 (LGR6) facilitates cell growth and migratory and invasive ability in GC through the PI3K/AKT/mTOR pathway [18, 19]. Liu et al. showed that Neuropilin-1 (NRP1) represses CD8+ T cell-mediated antitumor immune responses and anti-PD1 immunotherapy, leading to tumor relapse [20]. Moreover, NRP1 enhances metastasis through promoting epithelial-mesenchymal transition (EMT) in GC [21, 22]. The expression of Anosmin-1 (ANOS1), a secreted glycoprotein of about 100 kDa, in serum and tissues is a potential biomarker for diagnosing GC [23, 24]. ANOS1 was also reported to facilitate cell proliferation, migratory and invasive abilities of GC cells [24]. Increased expression of integrin subunit alpha V (ITGAV) significantly enhances cell proliferation and migration, leading to a poor prognosis [25]. It was found that fatty acid binding protein 3 (FABP3) regulates mitochondrial metabolism to promote tumor progression [26, 27]. It is well known that the immune response in the TME can profoundly affect the programmed death of tumor cells [28]. Cuproptosis, as a recently identified programmed cell death mechanism, its regulatory pathways and mechanisms by the immune system are also being explored. However, there is no research that explores the potential relationship between cuproptosis and immune-related genes in the GC TME. Therefore, we try to investigate its regulatory mechanism through bioinformatic analysis, helping establish the foundations for further research. In addition, fully exploring the relationship between cuproptosis and immune-related genes in TME is crucial for better understanding the progression of GC to design better individualized treatment strategies.

Here, we used bioinformatic analysis to comprehensively explore the profiles and prognostic values of cuproptosis-related immune genes (CRIGs) in GC. We established and validated a potent signature utilizing seven CRIGs to predict patient prognosis and further elucidated the intrinsic and potential regulatory network between these genes and cuproptosis in GC. Furthermore, the expression of seven CRIGs at the single-cell level in GC was also analyzed. In addition, we immunohistochemically validated the expression of NRP1, CXCR4, LGR6, CTLA4 and the key regulator of cuproptosis, ferredoxin 1 (FDX1), in nine paired tumor tissues and adjacent tissues. This research provides a potent signature toward accurately forecasting the long-term prognosis and efficacy of comprehensive therapies in GC patients.

## RESULTS

### Screening of differentially expressed CRIGs

**Figure 1:**
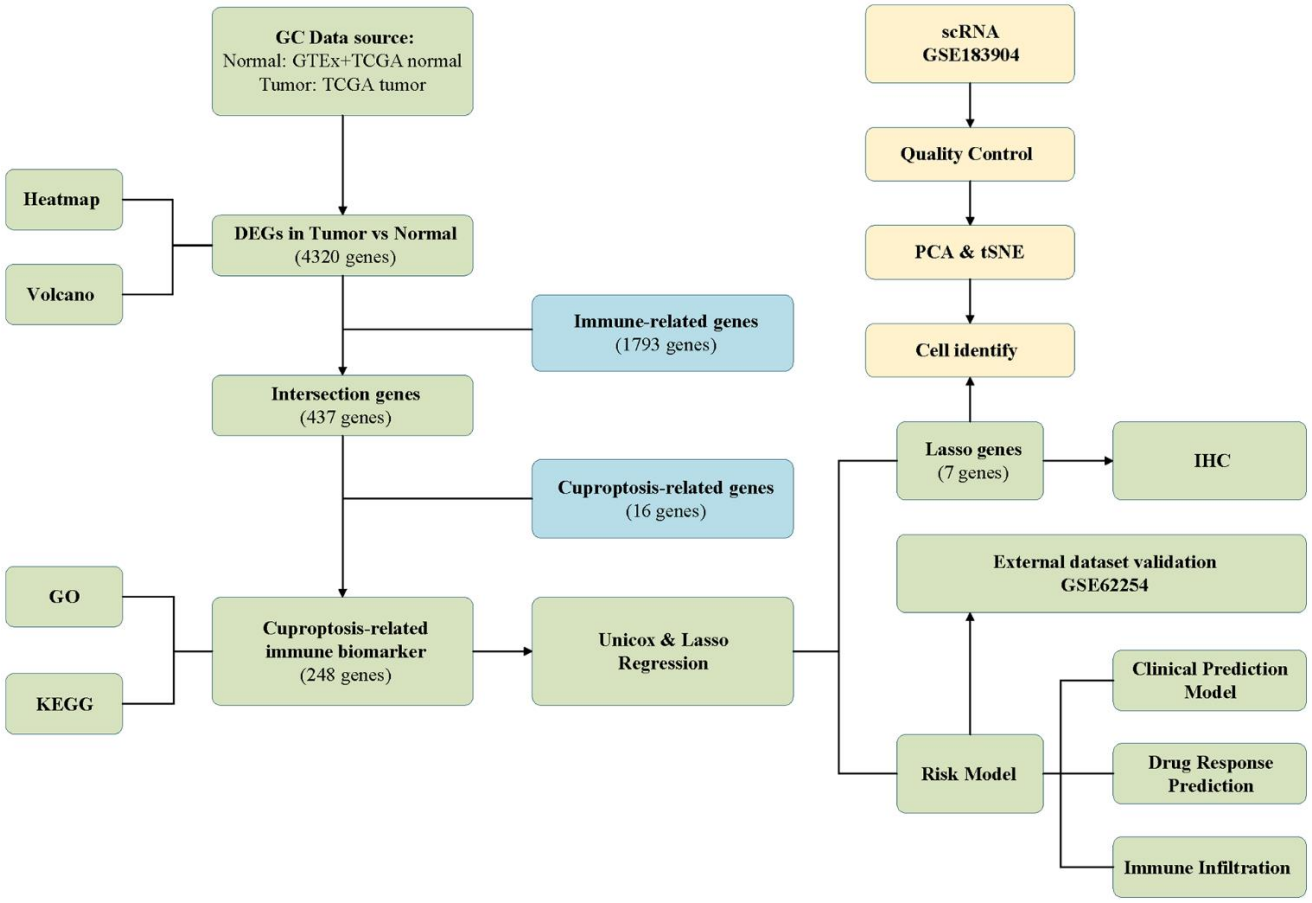
The analysis flow of this study.

**Figure 2:**
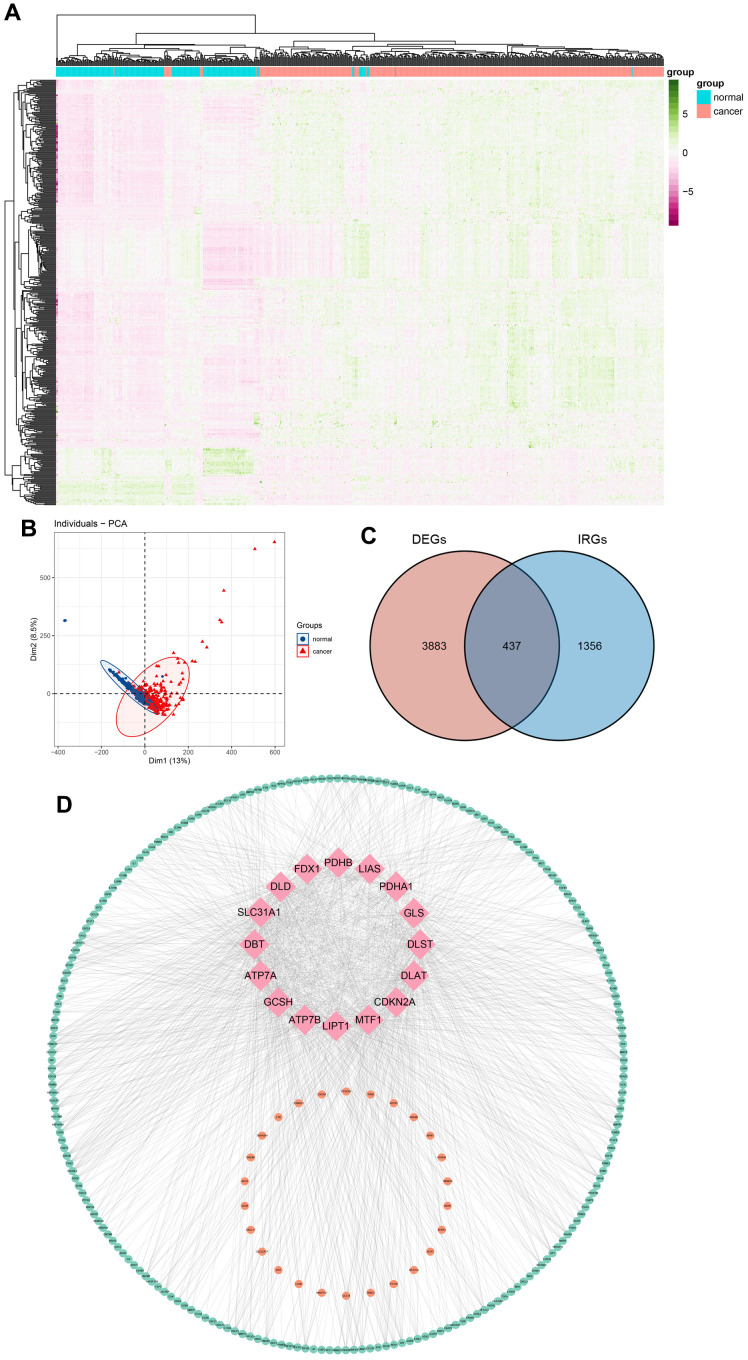
**Differentially expressed cuproptosis-related immune genes (CRIGs) in gastric cancer (GC).** (**A**) Heatmap of differentially expressed genes in GC. (**B**) Results of PCA analysis between GC and normal gastric tissues. (**C**) Venn diagram of differentially expressed genes and immune-related genes. (**D**) Correlation between immune-related genes and cuproptosis-related genes (pink, orange, and green represent cuproptosis-related genes, IRGs positively related to the cuproptosis and IRGs negatively related to the cuproptosis).

**Figure 3:**
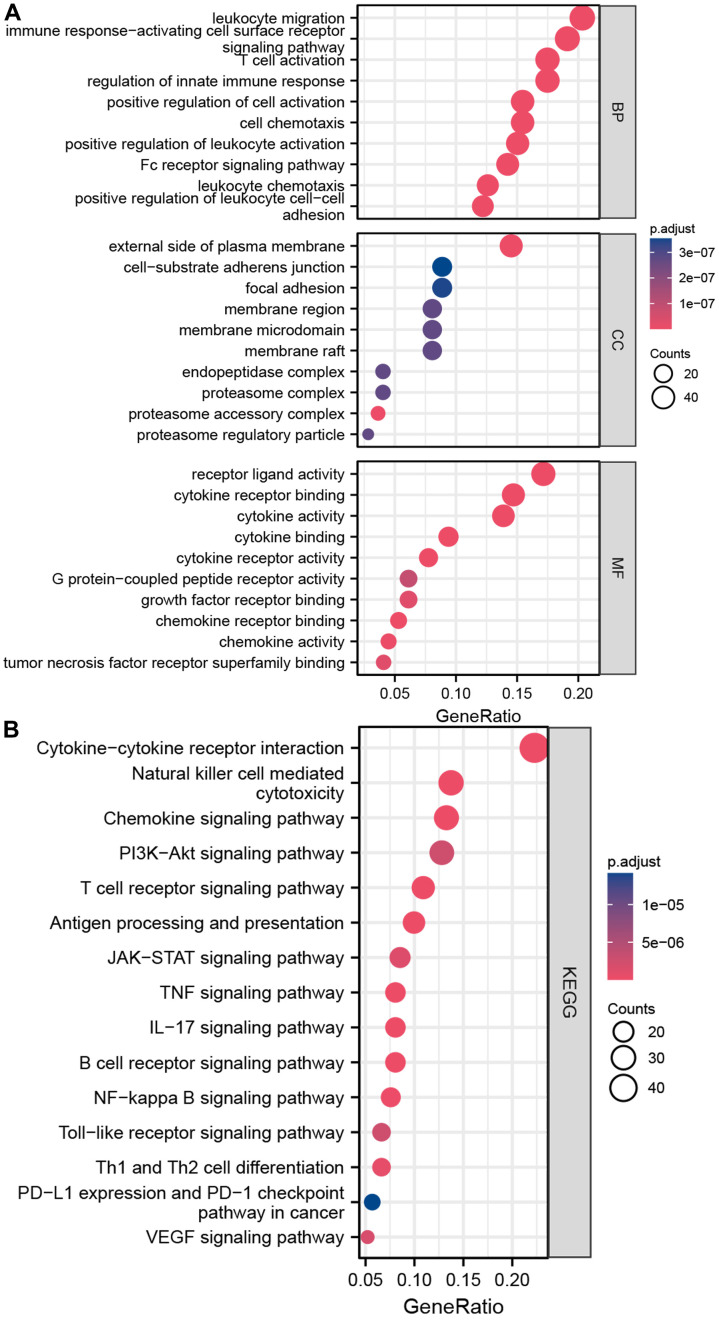
**Enrichment analyses of differentially expressed CRIGs.** (**A**) The results of GO analysis. (**B**) The results of KEGG analysis.

The schematic in Figure 1 shows the fundamental analysis flow of this research. A comprehensive and integrated analysis of the dysregulated genes in normal and GC tissues was performed to identify gene sets that have prognostic values for GC patients. This study’s data for normal tissues has two sources: the profiles of normal gastric mucosa obtained from the GTEx database and the profiles of paired adjacent tissues of tumors extracted from the TCGA database. The profiles of all the GC tissues were acquired from TCGA database. As shown in Figure 2A, the heatmap depicts the DEGs between normal and GC tissues. PCA revealed intrinsic differences in principal components between normal and GC samples (Figure 2B). Our findings confirmed the heterogeneity of tissue components between GC and normal gastric mucosa. The immune-related genes (IRGs) were extracted from the ImmPort database [29]. We found 437 IRGs (379 upregulated IRGs and 58 downregulated IRGs) that were significantly differentially expressed between normal and GC tissues (Figure 2C). We also identified 16 cuproptosis-related genes obtained from top published literature [9]. To investigate the potential correlation between IRGs and cuproptosis-related genes, the criteria of |cor| >0.2 and p<0.05 were set, which revealed 222 IRGs to be positively related to the cuproptosis, while 26 were negatively related (Figure 2D). We then explored the potential functions of differently expressed CRIGs by GO and KEGG analyses, wherein biological process analysis revealed that CRIGs are mainly involved in biological functions and signal transduction of immune cells. Cellular component analysis revealed that CRIGs are mainly located on the cell-substrate adherens junction, focal adhesion, and membrane region and external side of the plasma membrane (Figure 3A). Molecular function analysis found that CRIGs primarily participated in receptor-ligand activity, cytokine receptor binding, cytokine activity, and cytokine binding. The data of KEGG enrichment analysis showed the CRIGs to be mostly enriched in PI3K-Akt and other pathways to regulate tumor progression (Figure 3B). We preliminarily explored the decisive roles of CRIGs in GC through the abovementioned methods.

### Establishment and verification of the cuproptosis-related immune biomarker signature

**Figure 4:**
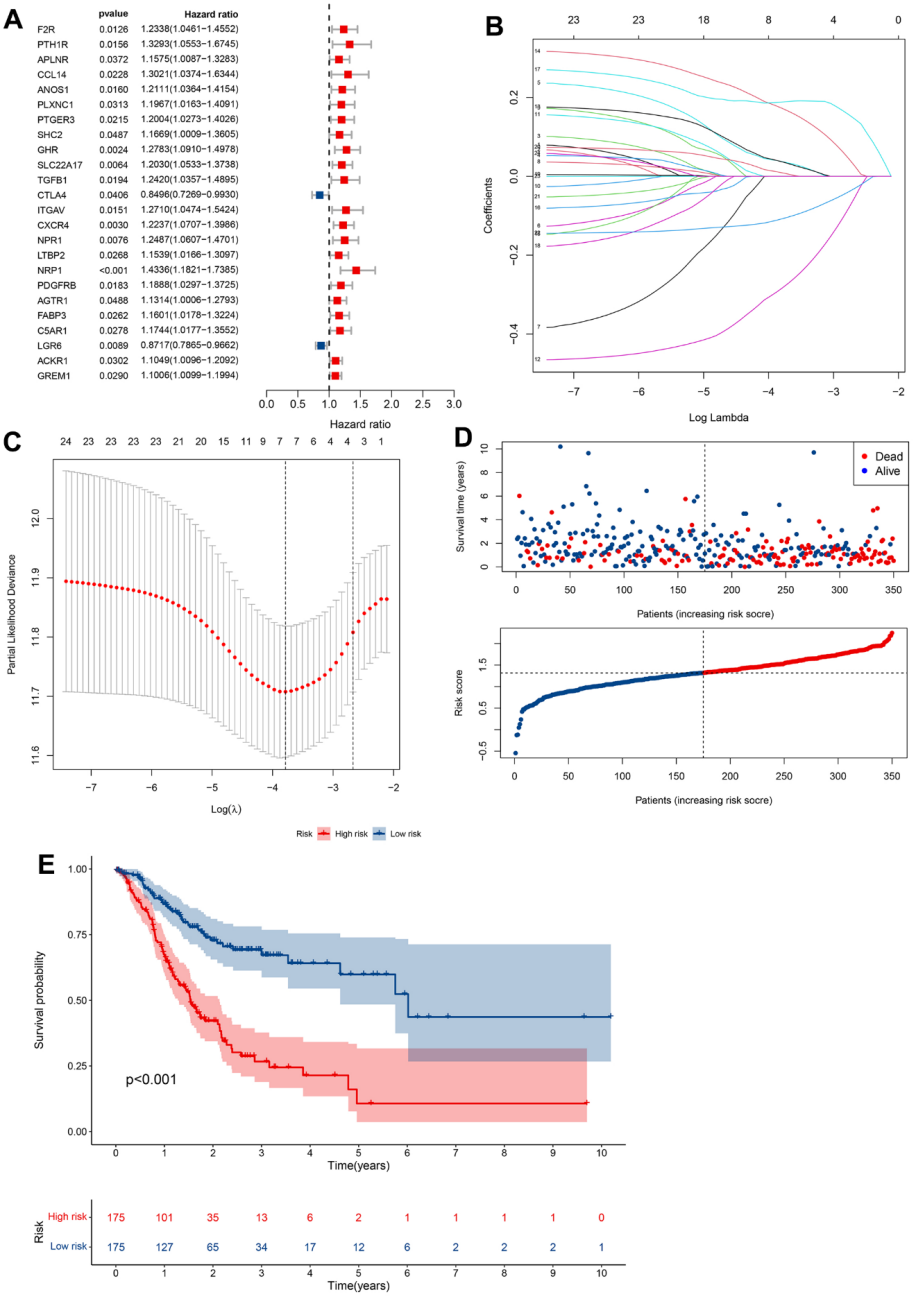
**Construction of prognostic models based on CRIGs.** (**A**) Identification of prognostic CRIGs for GC by univariate Cox regression analysis. (**B**) LASSO Cox regression analysis of the association between coefficients of genes and log(λ). (**C**) LASSO Cox regression analysis of the association between deviance and log(λ). (**D**) The survival status and survival time of GC patients ranked by risk score. (**E**) Kaplan-Meier analysis between high-risk groups and low-risk groups.

**Figure 5:**
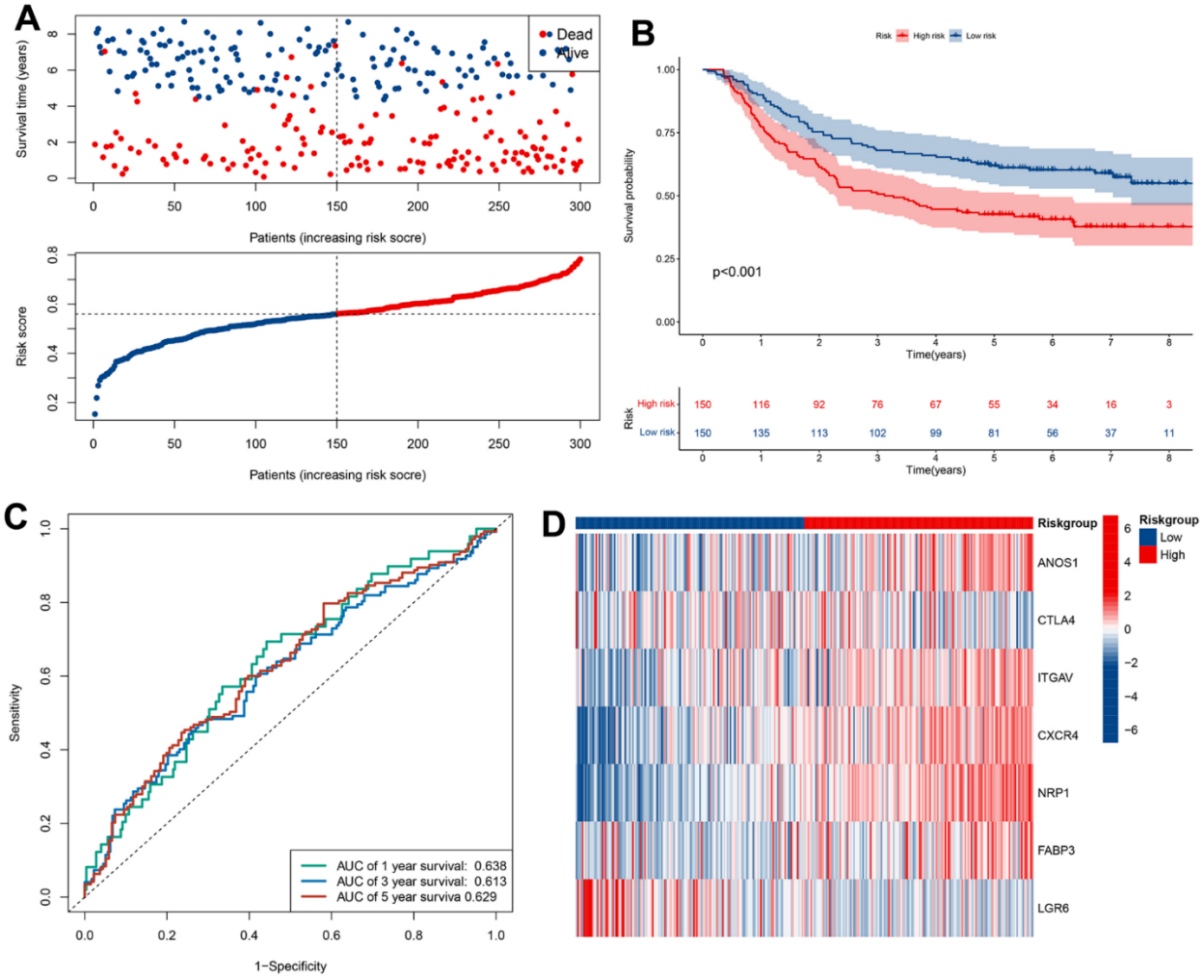
**Validation of prognostic models based on CRIGs in GSE62254 dataset.** (**A**) The survival status and survival time of GC patients ranked by risk score in GSE62254 dataset. (**B**) Kaplan-Meier analysis between high-risk groups and low-risk groups in GSE62254 dataset. (**C**) Time-dependent ROC curve of risk score predicting the overall survival (OS) in GSE62254 dataset. (**D**) Heatmap showed the differences of 7 CRIGs between high risk and low risk patients in GSE62254 dataset.

Our findings so far led us to perform univariate Cox analysis to investigate the predictive values of CRIGs for long-term survival in GC patients. The results revealed that 24 CRIGs had potential prognostic value in GC patients (Figure 4A), enabling us to establish a cuproptosis-related immune biomarker signature to predict the same using LASSO Cox regression analysis (Figure 4B, 4C). The risk signature comprised seven CRIGs (ANOS1, CTLA4, ITGAV, CXCR4, NRP1, FABP3, and LGR6) for which we calculated the risk scores: Risk score= (0.0373 × ANOS1) + (-0.2829 × CTLA4) + (0.0366 × ITGAV) + (0.1722 × CXCR4) + (0.1885 × NRP1) + (0.0088 × FABP3) + (-0.1075 × LGR6). Two groups of GC patients were distinguished based on the mean value of risk scores. The data revealed a higher proportion of patients who died and a lower long-term survival rate in the high-risk groups of GC (Figure 4D, 4E). These observations indicated that the cuproptosis-related immune biomarker signature efficiently predicted the prognosis in GC patients. We then used the same method to validate the accuracy of the cuproptosis-related immune biomarker signature identified from the GEO database, revealing a high consistency with the findings from TCGA dataset (Figure 5A, 5B). The data of ROC analysis revealed the AUC of 1, 3, and 5 years to be 0.638, 0.613, and 0.629 (Figure 5C). Furthermore, the heatmap presented the profiles of seven CRIGs in the two groups (Figure 5D). So far, we have established a cuproptosis- related immune biomarker signature to forecast the long-term survival rate of GC patients and verified its efficiency.

### The cuproptosis-related immune biomarker signature was a robust prognostic indicator for GC patients

**Figure 6:**
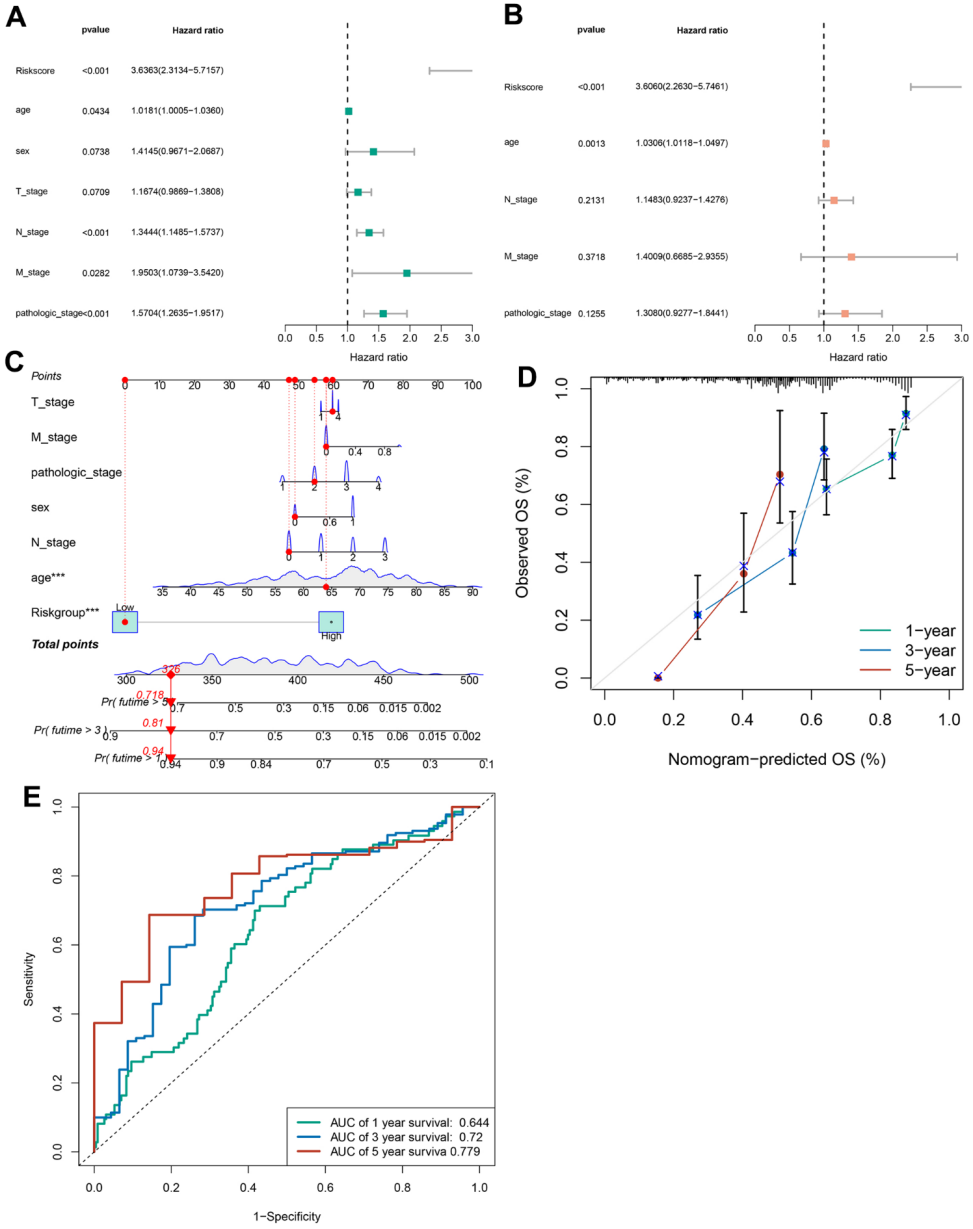
**Construction of a nomogram.** (**A**) The correlations between the risk score for OS and clinicopathological factors by univariate Cox regression analysis. (**B**) The correlations between the risk score for OS and clinicopathological factors by multivariate Cox regression analysis. (**C**) Details of the nomogram. (**D**) The calibration curve for predicting 1-, 3-, and 5-year OS. (**E**) Time-dependent ROC curve of risk score predicting the 1-, 3-, and 5-year OS in TCGA dataset.

Univariate and multivariate Cox analyses verified that cuproptosis-related immune biomarker signature was a potent prognostic indicator for GC patients. The data of univariate Cox analysis revealed that risk score, age, N, M, and pathologic stage were related to the long-term survival of GC patients (Figure 6A). Furthermore, we confirmed that risk score and age still profoundly affected the long-term survival of patients (Figure 6B). A nomogram composed of the T stage, M stage, pathologic stage, sex, N stage, age, and risk score (Figure 6C) was established to forecast the long-term survival of patients more intuitively. The data of calibration curve revealed the effectiveness and stability of predictive signature for patients’ long-term survival (Figure 6D). The AUC data revealed that the prediction accuracy of the signature for 1-, 3-, and 5-year survival rates of GC patients was 0.644, 0.72, and 0.779 (Figure 6E).

### Correlation between the signature and pathological characteristics in GC patients

**Figure 7:**
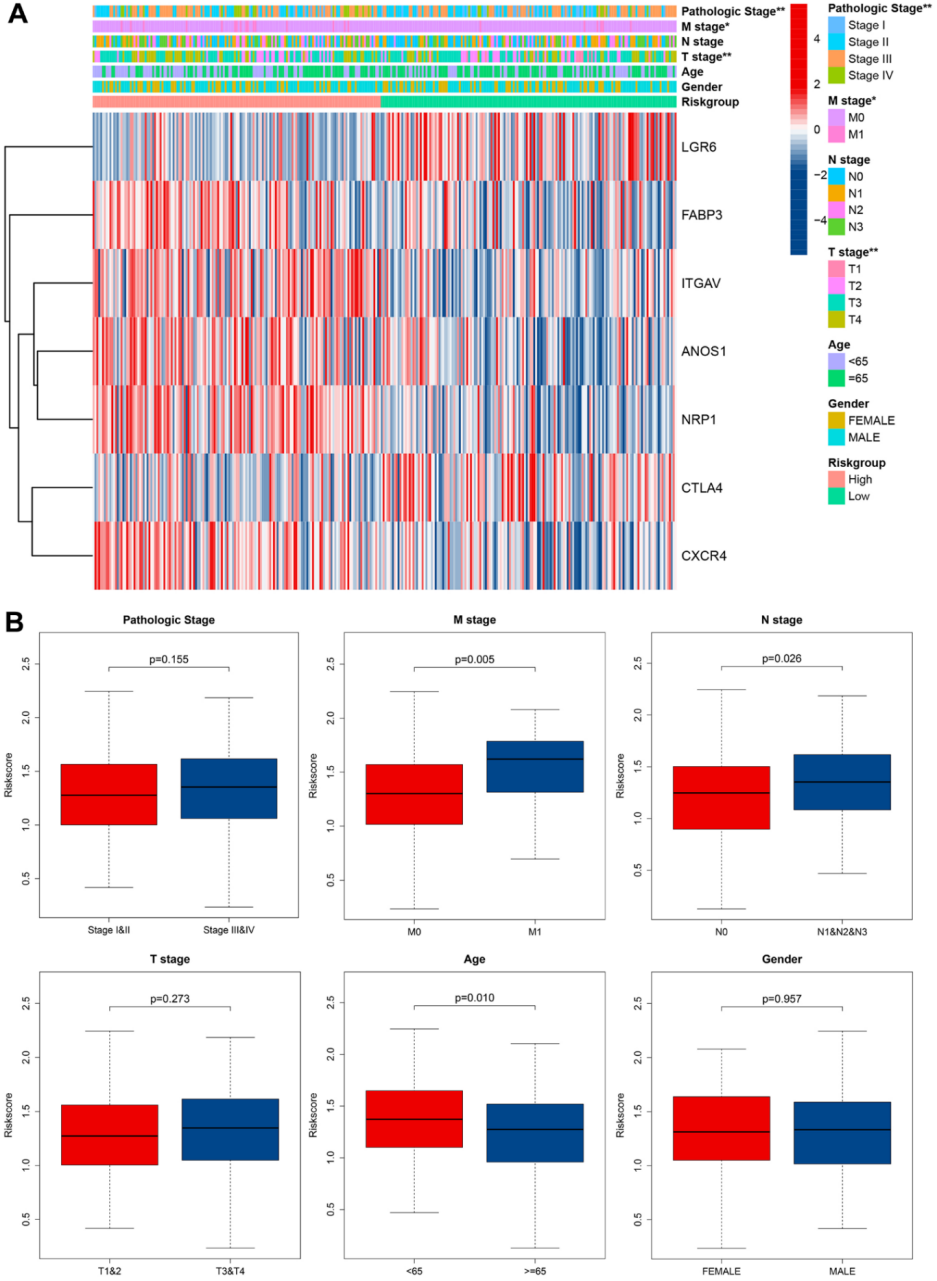
**Correlation between signature and clinical characteristics.** (**A**) Heatmap showed the differences of 7 CRIGs between high risk and low risk patients in clinical characteristics. (**B**) The risk score differences between I and II pathological stage and III and IV pathological stage, M0 stage and M1 stage, N0 stage and N1 and N2 and N3 stage, T 1 and 2 stage and T 3 and 4 stage, age under 65 and age over 65, male and female.

We assessed the relationship between the cuproptosis-related immune biomarker signature and pathological characteristics in GC patients using chi-square test (Figure 7A). Our analysis revealed that the T, N, M, and pathological stages were more malignant in the GC patients with high risk scores. Additionally, the risk scores for the M1 stage, N1 and N2 and N3 stage, and age groups under 65 years were higher than those for the M0 stage, N0 stage, and age groups over 65. In contrast, the risk scores of the different pathological stages, T stage, and gender in GC patients were no significance (Figure 7B). These findings suggested that the prognostic model not only accurately predicted GC patients’ prognosis but may also indicated their clinicopathological characteristics. Stratification analysis further investigated the efficiency of the signature in subgroups, revealing that age >= 65, age < 65, female, male, M1 stage, M0 stage, N0 stage, N1 and N2 and N3 stage, Stage I-II, Stage III-IV, T1-T2 stage, and T3-T4 stage, GC patients with high risk scores often owned a poor long-term survival (Supplementary Figure 1).

### Functional enrichment analyses and GSEA

**Figure 8:**
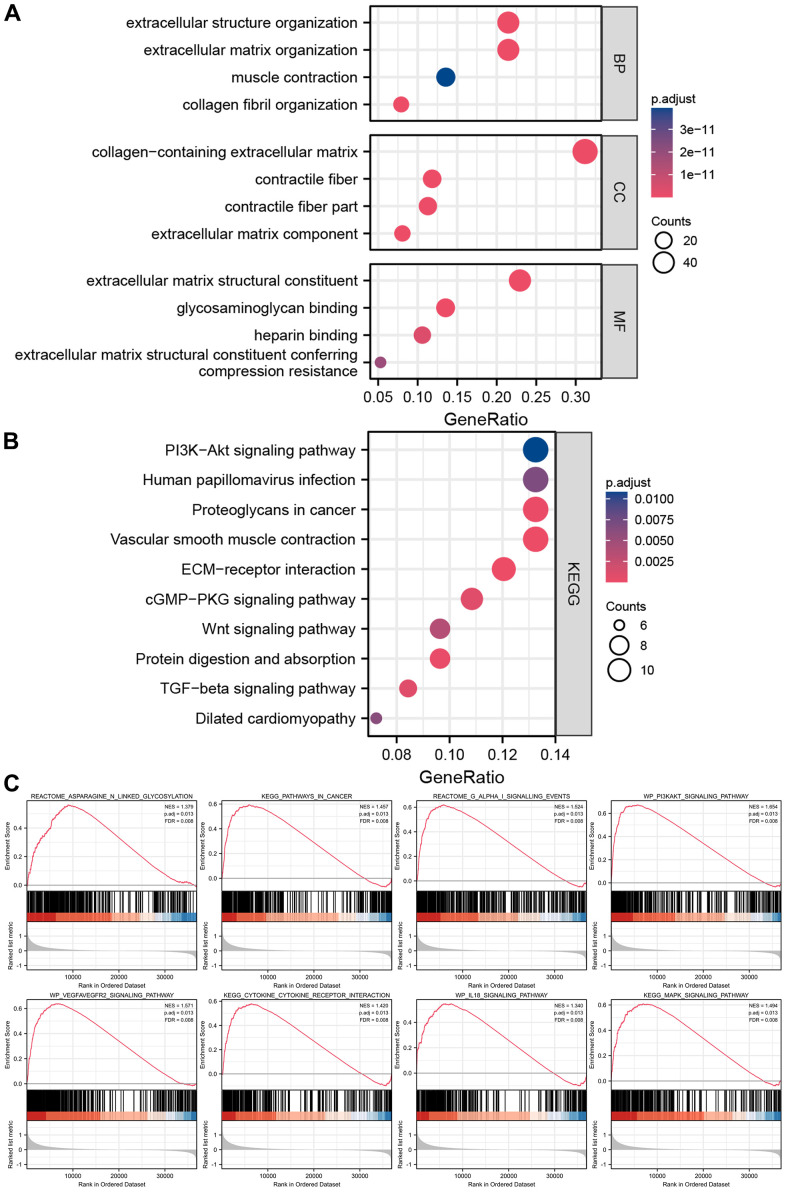
**Enrichment analyses and GSEA analysis of differentially expressed genes between high-risk groups and low-risk groups.** (**A**) The results of GO analysis. (**B**) The results of KEGG analysis. (**C**) The results of GSEA analysis between high-risk groups and low-risk groups.

GO and KEGG analyses were utilized to investigate the latent biological roles of DEGs in two groups. As shown in Figure 8A, the DEGs were mostly involved in processes like muscle contraction and other processes. Cellular component analysis revealed the DEGs to be mainly located in the collagen-containing extracellular matrix, contractile fiber, and extracellular matrix components. Molecular function analysis revealed that the DEGs especially participated in intercellular communications. KEGG analysis revealed the DEGs have significant enrichment of the PI3K-Akt pathway, proteoglycans in cancer, ECM-receptor interaction, and cGMP-PKG pathway to modulate tumor progression (Figure 8B). GSEA analysis clarified the molecular mechanisms enriched or depleted in the two groups in the cuproptosis-related immune biomarker signature. Figure 8C shows a significant enrichment of asparagine N-linked glycosylation, pathways in cancer, G alpha (i) signalling events, PI3K-Akt, VEGFA-VEGFR2, IL-18, and MAPK signaling pathway, and cytokine-cytokine receptor interaction in high-risk groups of GC patients.

### Analyzing the level of immune infiltration of the cuproptosis-related immune biomarker signature

**Figure 9:**
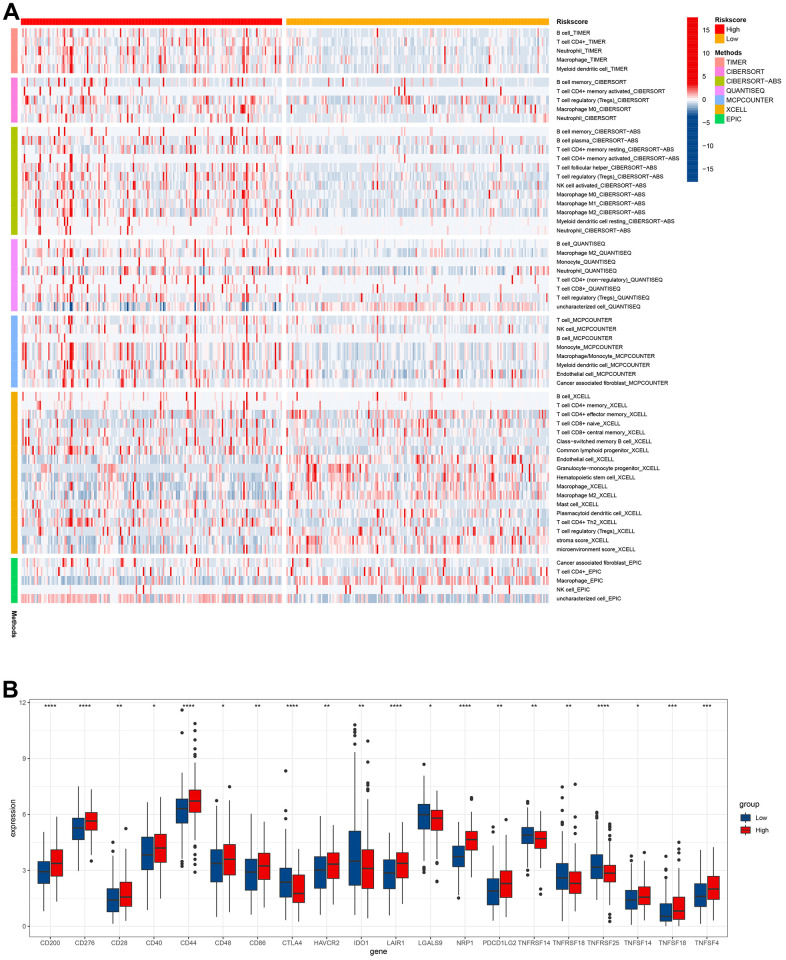
**Immune infiltration level analysis based on the risk model.** (**A**) Immune cells infiltration between high-risk groups and low-risk groups. (**B**) The expression of immune checkpoints between high-risk groups and low-risk groups.

Heatmap revealed the correlation between cuproptosis-related immune biomarker signature and immune infiltration by analyzing data from the databases (Figure 9A). TIMER and CIBERSORT revealed that GC patients in high-risk groups presented a positive relation to infiltrating B cells, CD4^+^ T cells, neutrophils, macrophages, Tregs, myeloid dendritic cells, and M0 macrophages in the TME. Moreover, similar results for immune infiltration were obtained from CIBERSORTABS, QUANTISEQ, MCP-counter, XCELL, and EPIC databases. Moreover, the correlation of the risk model with immune checkpoint molecules was explored to enable a more accurate formulation of precise clinical therapy, given the emerging key role of immune checkpoint inhibitor therapy in the comprehensive treatment of GC. CD200, CD276, CD28, CD40, CD44, CD48, CD86 HAVCR2, LAIR1, NRP1, PDCD1LG2, TNFSF14, TNFSF18, and TNFSF4 were elevated in high-risk groups, while CTLA4, IDO1, LGALS9, TNFRSF14, TNFRSF18, and TNFRSF25 were decreased in high-risk groups (Figure 9B and Supplementary Figure 2). The above data would aid the rational selection of immune checkpoint inhibitors in treating GC patients.

### Drug sensitivity analysis

**Figure 10:**
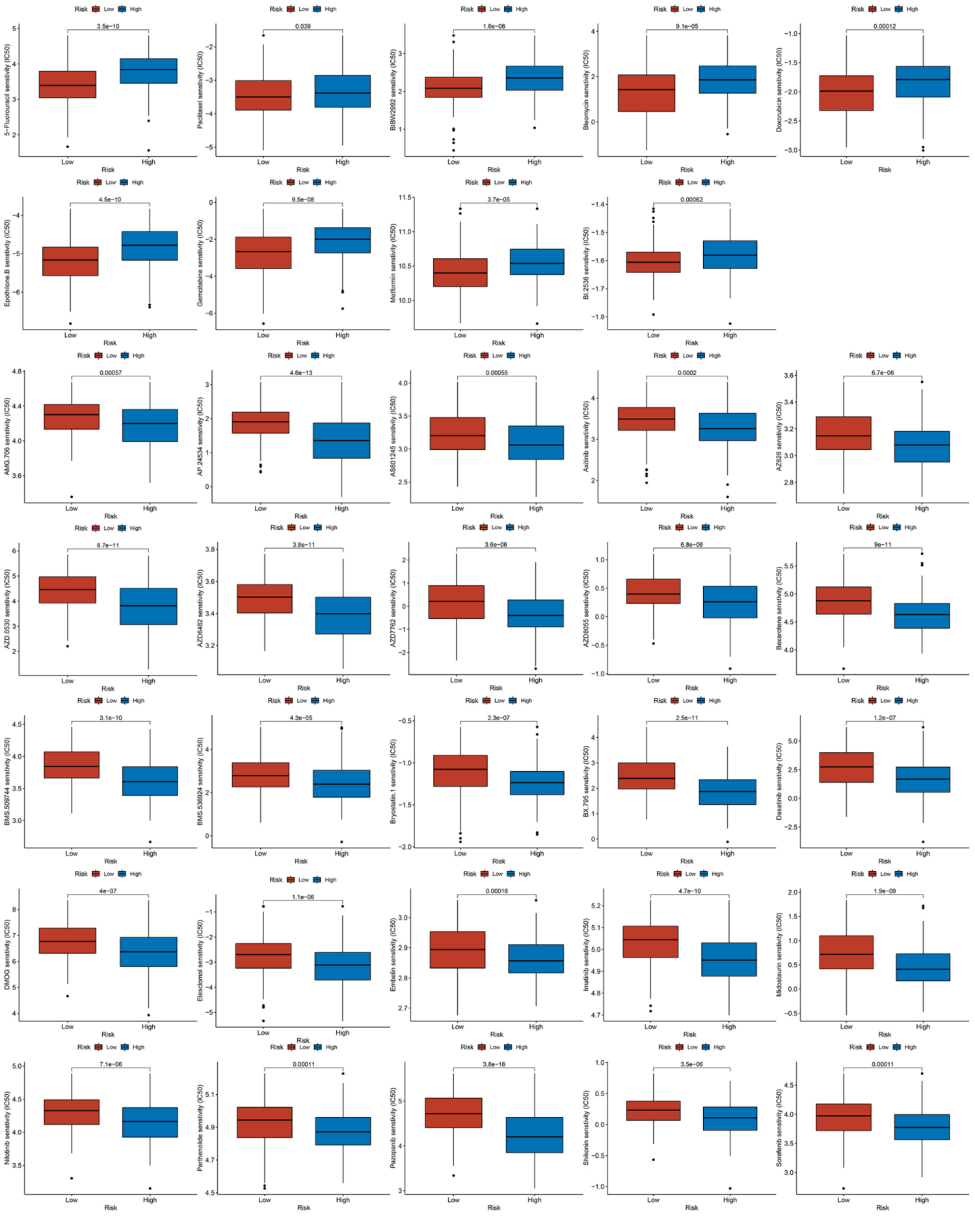
The results of drug sensitivity analysis between high-risk groups and low-risk groups.

Due to atypical early GC symptoms, more than 80 % of hospitalized patients are initially diagnosed with locally advanced or metastatic GC in China. This phenomenon warranted postoperative adjuvant chemotherapy for the treatment. This observation led us to analyze the chemosensitivity in two groups of GC patients. There was a significant difference in the half maximal inhibitory concentration of classic chemotherapy drugs between the two groups. GC patients with high risk scores were often resistant to 5-Fluorouracil and paclitaxel, suggesting the requirement for more appropriate drug selection and combination of chemotherapy regimens for them (Figure 10).

### The results of CRIGs in GC scRNA-seq

**Figure 11:**
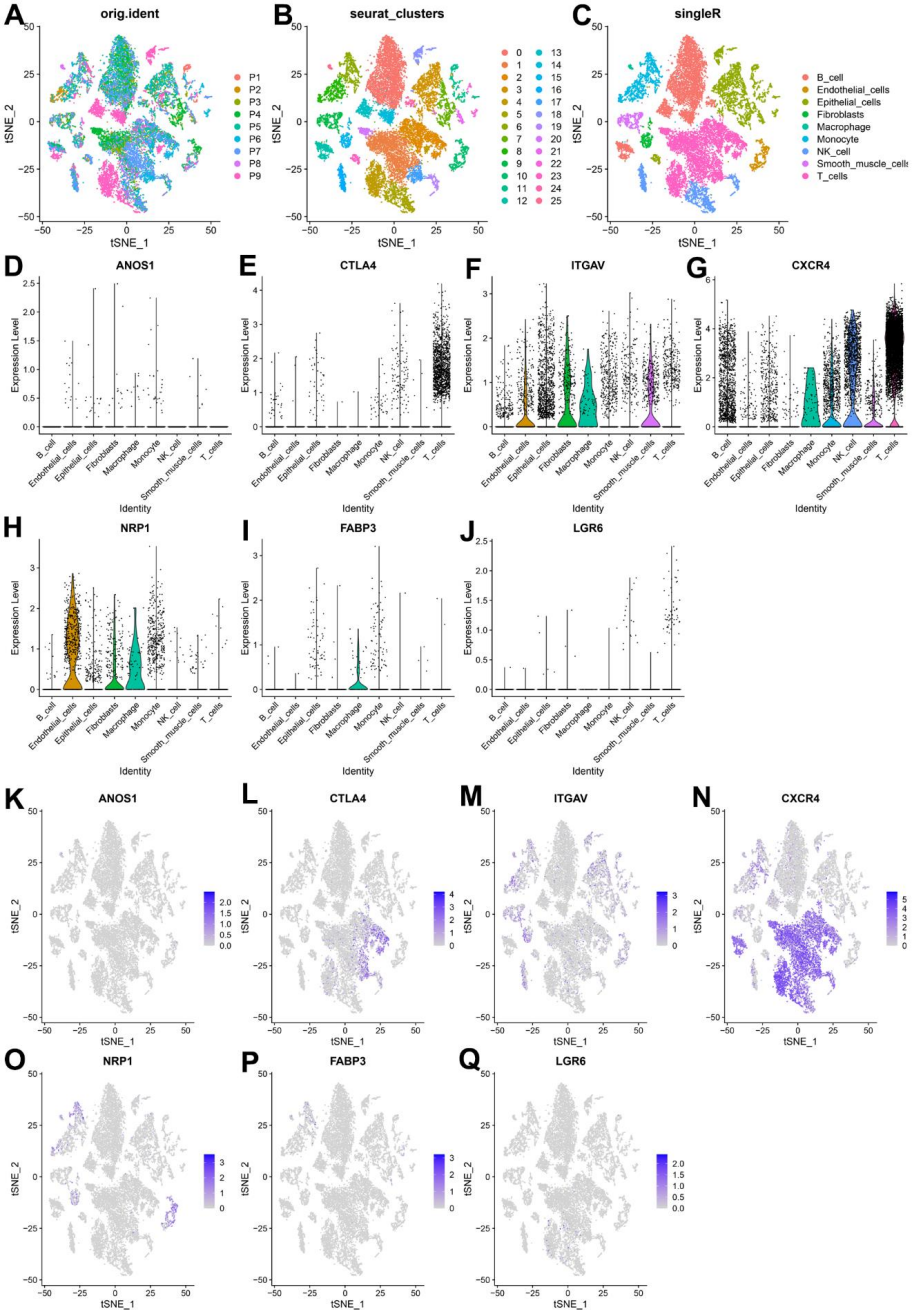
**Expression of CRIGs ANOS1, CTLA4, ITGAV, CXCR4, NRP1, FABP3 and LGR6 in GC single cells.** (**A**) Cell t-SNE distribution among different samples. (**B**) T-SNE distribution of 25 independent cell clusters. (**C**) T-SNE distribution of cell types after SingleR annotation. (**D**–**J**) Violin plots of ANOS1, CTLA4, ITGAV, CXCR4, NRP1, FABP3 and LGR6 abundance in GC at Single Cell Level. (**K**–**Q**) The t-SNE distribution of ANOS1, CTLA4, ITGAV, CXCR4, NRP1, FABP3 and LGR6 abundance in GC at Single Cell Level.

We extracted and analyzed nine specimens of GC scRNA-seq from the GSE183904 dataset, and performed quality control of the data. 20859 cells were obtained after filtering the cells with mitochondrial gene content > 25 %, the nFeature RNA < 100, and nFeature RNA > 5000 criterions. The quality control indicators such as the number of genes, unique molecular identifiers (UMI), and mitochondrial gene ratio before and after quality control were displayed (Supplementary Figure 3A–3K). 3,000 variable features of the dataset are detected using the ‘vst’ method by employing the ‘FindVariableFeatures’ function, dimensioned and visualized using PCA analysis (Supplementary Figure 3L). By selecting the first 35 principal components as the statistically significant input of t-SNE (Supplementary Figure 3M), the cells were classified into 25 independent clusters (Figure 11A, 11B). SingleR was used to identify the cell clusters as 9 cell types: B cells, endothelial cells, epithelial cells, fibroblasts, macrophage, monocyte, NK cells, smooth muscle cells, and T cells (Figure 11C). We investigated the expression of seven CRIGs (ANOS1, CTLA4, ITGAV, CXCR4, NRP1, FABP3, and LGR6) at the single cell level of GC (Figure 11D–11Q). The data revealed that CTLA4 was only enriched in T cells. ITGAV was significantly enriched in endothelial cells, epithelial cells, fibroblasts, and macrophage cells. CXCR4 was widely enriched in B cells, macrophages, monocytes, NK cells, and T cells. NRP1 was detected to be dramatically enriched in endothelial cells, fibroblasts, and macrophages. The abovementioned results indicated that CRIGs in the specific cell types played key roles in the GC progression.

### The confirmation of cuproptosis-related immune biomarkers in GC specimens

**Figure 12:**
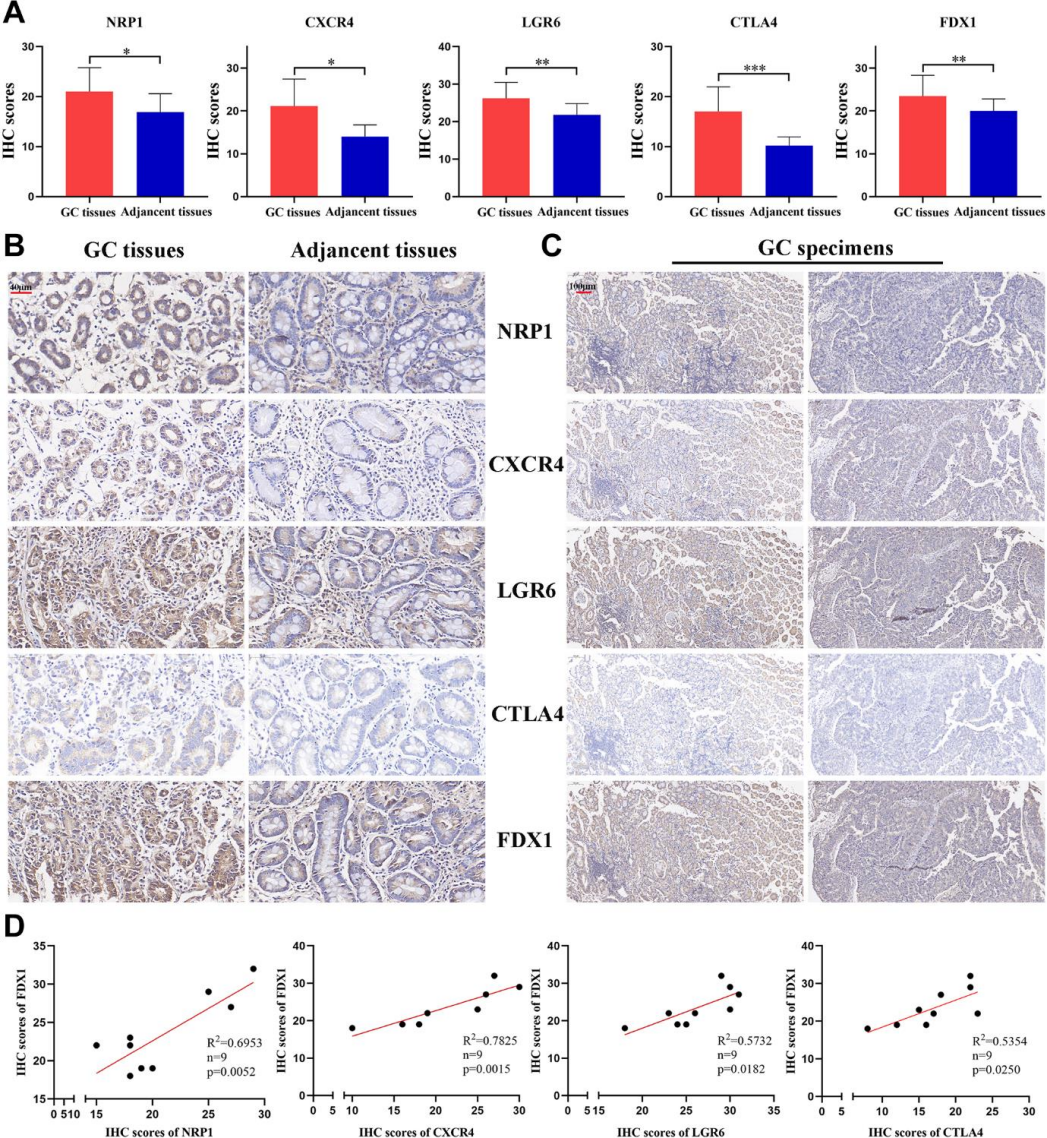
**The expression of 4 CRIGs and FDX1 in GC.** (**A**) The IHC scores of NRP1, CXCR4, LGR6, CTLA4, and FDX1 in GC tissues and adjacent normal tissues. (**B**) The representative IHC images of NRP1, CXCR4, LGR6, CTLA4, and FDX1 in GC tissues and adjacent normal tissues (scale bar: 40 μm). (**C**) The representative IHC images of NRP1, CXCR4, LGR6, CTLA4, and FDX1 in the same GC tissues (scale bar: 100 μm). (**D**) The correlation between NRP1, CXCR4, LGR6, CTLA4 and FDX1 in the GC tissues. Data represent the mean ± SD. Student’s t-test was used to determine statistical significance: *p < 0.05, **p < 0.01, ***p < 0.001.

We selected four genes from the cuproptosis-related immune biomarkers and FDX1 for validation via immunohistochemistry in GC samples, which revealed NRP1, CXCR4, LGR6, CTLA4, and FDX1 to be elevated in the GC tissues in comparison with adjacent tissues (Figures 12A, 12B), validating our data in the abovementioned database analysis. Moreover, IHC of the same tissue separately revealed that the expression of FDX1 increased with an elevation in NRP1, CXCR4, LGR6, or CTLA4 (Figure 12C). Furthermore, Pearson regression analysis demonstrated that the expression of NRP1, CXCR4, LGR6, and CTLA4 have a positive relation to FDX1 in nine GC tissues (Figure 12D). The above results validated potential and intrinsic connections between the immune microenvironment and cuproptosis in GC, that is, the immune response in TME affected the progression of GC by regulating the cuproptosis process of tumor cells.

## DISCUSSION

GC ranks third in terms of morbidity and mortality in China, according to the latest cancer statistics by the National Cancer Center in 2022, which shows about 80 % of GC patients being diagnosed at advanced stage, with a dismal long-term survival [2, 30]. Despite the great development of comprehensive therapies, GC patients with locally advanced disease showed a dismal long-term survival rate of as low as 20 to 30% [31]. Fortunately, the emergence of immune checkpoint inhibitors has brought the dawn on treating advanced-stage cancer patients. It is also the first line of therapy for GC patients with advanced stage, significantly prolonging their long-term survival [32, 33]. Tsvetkov et al. reported that cuproptosis, a new type of copper-dependent programmed cell death, may significantly affect the uncontrolled cell proliferation of tumors [9]. Immune microenvironment may be closely and intrinsically associated with cell cuproptosis in tumors [10, 11]. However, the connection between cell cuproptosis and immune regulatory factors remains unclear. The abovementioned facts indicate great clinical significance in exploring the intrinsic mechanism of immunoregulatory factors and cell cuproptosis to identify robust potential prognostic biomarkers in GC.

Differentially expressed IRGs (437) between GC and normal gastric mucosa tissues from TCGA and GTEx databases were identified and were first chosen for this investigation. Then, in a preliminarily screening using correlation analysis, we discriminated 26 IRGs adversely correlated to and 222 IRGs positively associated with the cuproptosis. Using functional enrichment analysis, we identified the putative mechanism underlying the functions of the differentially expressed CRIGs in GC patients. The differentially expressed CRIGs were enriched for classical cytokine-cytokine receptor interactions and biological functions of other immune cells, along with the PI3K-Akt, JAK-STAT, and TNF signaling pathways. Xu et al. found that PI3K-Akt pathway regulated GC progression by restraining the PD-1/PD-L1-dependent T cell immunization [34]. It has also been shown that the JAK-STAT pathway influences PD-L1 expression and immune evasion through interferon γ in GC [35]. In addition, TNF-α signaling pathways promote PD-L1 expression and immunosuppression in GC cells [36, 37]. Subsequently, we used univariate Cox and LASSO analyses to establish a risk signature consisting of seven CRIGs. Next, two groups of GC patients were discriminated by the mean value of all patients’ risk scores. KM analysis presented that GC patients with low risk scores often had lower mortality and longer overall survival. Furthermore, the test dataset verified the reliability and efficiency of the risk signature in prognosis prediction. The low prognostic accuracy of the TNM staging, the currently and generally used technique to forecast the long-term survival of GC patients [3], led us to investigate whether the risk model could supplement them. The data confirmed that our risk signature was a potent biomarker for predicting the long-term survival of GC patients. A nomogram consisting primarily of TNM staging and risk model led to an increase in the predictive accuracy of GC patients’ prognosis. The risk signature alone predicted the long-term survival of GC patients for 1-, 3- and 5-year survival with an accuracy of 0.644, 0.72, and 0.779, respectively. Moreover, GC patients with high risk scores often had advanced TNM staging, while those with low risk scores tended to have early TNM staging. We also compared the putative functional roles of the DEGs in the two groups. Similarly, we identified the well-studied PI3K-Akt pathway to act pivotal roles in the malignant development of GC. The GSEA results proved that asparagine N-linked glycosylation, pathways in cancer, G alpha (i) signalling events, PI3K-Akt pathway, VEGFA-VEGFR2 and other pathways were significantly gathered in GC patients of the high-risk groups.

M2 tumor-associated macrophages (TAMs) promote tumor progression by producing immunosuppressive cytokines such as IL-10 and TGF-β [38]. This observation corroborates our findings that the quantities of M2 TAMs in the patients with high risk scores were more than that in the patients with low risk scores. CD8^+^ T cells, a vital component of the adaptive immune system, play crucial roles in clearing tumor cells [39]. Our study revealed that GC patients had less CD8^+^ T cell infiltration, resulting in shortening their survival in high-risk group. Activated regulatory T cells (Tregs) exert immunosuppressive functions by inhibiting the activation and proliferation of CD4^+^ CD25^−^ and CD8^+^ T cells [40]. GC patients with high risk scores usually had a higher proportion of Tregs, indicating a poor long-term survival. Neoadjuvant therapy and postoperative chemotherapy are crucial for advanced-stage GC patients. Drug sensitivity estimation to traditional chemotherapy regimens showed that GC patients were usually more resistant to 5-Fluorouracil and paclitaxel in the high-risk group, suggesting the requirement for more appropriate drug selection and combination of chemotherapy regimens for them. Fortunately, the emergence of immune checkpoint inhibitors has brought hope for treating patients with advanced GC. Our investigation found that most of the critical immune checkpoints were lower in the GC patients with high risk scores. The abovementioned findings proved cuproptosis-related immune biomarker signature to be a valuable reference for selecting comprehensive treatment options for individuals with advanced GC. scRNA-seq is widely used to investigate the genes profiles at the single-cell level. It can more accurately detect the levels of gene expression in specific cell types and reveal cell heterogeneity in the TME [41]. The expression of seven CRIGs (ANOS1, CTLA4, ITGAV, CXCR4, NRP1, FABP3, and LGR6) was investigated at the single cell level of GC. The data revealed that CTLA4 was only enriched in T cells. ITGAV was significantly enriched in endothelial cells, epithelial cells, fibroblasts, and macrophage cells. CXCR4 was widely enriched in immune cells. NRP1 was detected to be dramatically enriched in endothelial cells, fibroblasts, and macrophages. This data revealed that CRIGs in the specific cell types played key roles in the GC progression. We validated the elevated expression of NRP1, CXCR4, LGR6, CTLA4, and FDX1 in GC tissues compared with paired adjacent tissues via IHC to preliminarily test the cuproptosis-related immune biomarker signature. Our investigation revealed positive correlations between NRP1, CXCR4, LGR6, CTLA4, and FDX1 in the GC tissues. However, more in-depth experiments *in vitro* and *in vivo* and specific mechanistic investigations are warranted to investigate the correlation between immune infiltration in TME and cell cuproptosis in GC.

To conclude, we established a novel signature utilizing seven differentially expressed CRIGs for forecasting the long-term survival of GC patients. Our risk signature showed good clinical practicability for forecasting the long-term survival of GC patients. Based on our findings, we propose that this signature can efficiently complement the choice of personalized precision therapy for GC patients.

## MATERIALS AND METHODS

### Tissue specimen

Nine paired GC tissues and corresponding adjacent tissues were obtained from the Department of General Surgery, Peking Union Medical College Hospital (PUMCH). We ensured that all enrolled GC patients did not receive any preoperative treatments. Specimens were thoroughly soaked in liquid formaldehyde and fixed for immunohistochemistry.

### Data collection and identification of differentially expressed CRIGs

Raw data of gene transcriptome profiles of gastric cancer and normal gastric specimens were collected from TCGA, GTEx, and GEO database. The data from TCGA (414 GC specimens and 36 normal gastric specimens) and GTEx (174 normal gastric specimens) were acquired from UCSC Xena [42], a genetic data analysis platform, standardized and then combined for the representational difference analysis (RDA). Acquisition of cuproptosis-related genes was from the first published literature on cell cuproptosis [9]. The immune-related genes (IRGs) used for this study were extracted from the ImmPort database [29]. Moreover, the prognostic accuracy of the cuproptosis-related immune biomarker signature was verified by GSE62254 containing 300 GC samples and the corresponding clinical and prognostic information [43].

### Functional enrichment analyses

The differentially expressed genes (DEGs) were analyzed by gene ontology (GO) [44] analysis. The Kyoto Encyclopedia of Genes and Genomes (KEGG) [45] analysis was used to investigate underlying molecular mechanisms of DEGs using the ClusterProfiler package [46].

### Establishment and verification of a prognostic signature using CRIGs

Univariate Cox regression analysis and least absolute shrinkage and selection operator (LASSO) Cox regression analysis were used to optimize and construct the prognostic risk signature of CRIGs. The calculation formula of risk score is: risk score = $\Sigma_{1}^{n}\text{Coef}\;\text{n}*\text{expression of mRNA}\;\text{n}$ Expression of mRNA n and Coef n mean expression of gene and regression coefficients, respectively. Subsequently, GC patients were separated into high- and low-risk groups based on the mean of the calculated risk scores. The survival differences in two groups were evaluated by Kaplan-Meier (KM) analysis. In addition, the validity of the risk signature in forecasting the long-term survival of GC patients was evaluated using ROC analysis. The GSE62254 dataset was used to verify the validity of the cuproptosis-related immune biomarker signature.

### Establish a nomogram

The independent predictive long-term survival ability of the signature for GC patients was tested using Univariate and multivariate Cox analyses. The cuproptosis-related immune biomarker signature and clinicopathological characteristics were considered to create a nomogram assessing the long-term prognosis in GC patients. The nomogram’s predictive power was evaluated using concordance index and calibration curve. In addition, we also investigated the connection between the clinicopathological characteristics and cuproptosis-related immune biomarker signatures.

### Immune cell infiltration analysis

Much research has verified the profound effects of immune infiltration in the TME on tumor progression. Therefore, level of immune infiltration was evaluated using TIMER, CIBERSORT [47], CIBERSORT-ABS, QUANTISEQ, MCPCOUNTER, XCELL, and EPIC datasets. The expression of several critical immune checkpoints in the two groups was also investigated.

### Drug sensitivity analysis

The analytical package (pRRophetic) [48] was applied to the Genomics of Drug Sensitivity in Cancer database (GDSC) to predict the response to chemotherapeutic drugs to investigate the differences in sensitivity towards them between the two groups.

### Single-cell RNA sequencing (scRNA-seq)

In this study, the scRNA-seq dataset of GC (GSE183904) was extracted from GEO database for analysis, and the detection platform was Illumina NovaSeq 6000. A total of nine GC specimens were used for analysis. Seurat (version 4.0) [49] is used for this Seurat object analysis. When a cell’s mitochondrial gene accounts for the highest proportion of all genes, it may be in a stress state. Therefore, cells with mitochondrial gene content greater than 25 % will be filtered. Since low-quality cells or empty droplets typically contain few genes, while double cells exhibit a high content of genes, the nFeature RNA < 100 and nFeature RNA > 5000 criterions are used to filter low-quality cells. Next, the ‘NormalizeData’ function is used to standardize the GSE173682 dataset. The standardization method is the ‘LogNormalize’ method, and the ‘vst’ method is used to detect 3000 variable features of the GSE173682 dataset by using the ‘FindVariableFeatures’ function. The ‘ScaleData’ function is used to scale the data to exclude the influence of different cell sequencing depth. Principal component analysis (PCA) is used to determine significance in principal components between tissues or cells through using the ElbowPlot function. The first 35 principal components are statistically significant inputs (dims = 35) as t-Distributed Stochastic Neighbor Embedding (t-SNE). The FindClusters function is used for cell clustering and cell type identification. In order to verify our cell type annotation, the HumanPrimaryCellAtlasData [50] dataset in the SingleR (version 1.8.1) [51] R package is used for standard cell type annotation.

### Immunohistochemistry (IHC)

The detailed procedures followed for IHC were consistent with the previous descriptions [52, 53]. The information of primary antibodies is listed as follows: CTLA4 (Cell Signaling Technology, 53560S), CXCR4 (Proteintech, 60042-1-Ig), NRP1 (Proteintech, 60067-1-Ig), LGR6 (Proteintech, 17658-1-AP), and FDX1 (Proteintech, 12592-1-AP). The use and dilution ratio of primary antibodies were in accordance with the manufacturers’ instructions. Five fields were randomly selected under the 40× objective lens. The IHC scores of the samples were calculated as the sum of the scores obtained for five fields. The staining intensities and positive percentages were included in the IHC scores. The degree of staining was rated as 0, 1, 2, and 3 points, corresponding to undetected, mildly stained, moderately stained, and strongly stained, respectively. Likewise, the percentage of positivity was divided into < 5 %, from 5 % to 25 %, from 26 % to 50 %, from 51 % to 75 %, and > 75 % of positively stained cells corresponding to 0, 1, 2, 3, and 4, respectively.

### Statistics analysis

All statistical analyses were conducted by the R software (version 4.1.3), SPSS 22.0 (Chicago, USA), and Graphpad Prism 8.0 (CA, USA). The differences in the two groups were compared using the Wilcoxon test. Statistics analysis was significant when *p* < 0.05.

## Supplementary Material

Supplementary Figures
