# Supplementary material for: Comprehensive analysis of cuproptosis-related immune biomarker signature to enhance prognostic accuracy in gastric cancer
Source: Aging (Albany NY). 2023 Apr 7;15(7):2772–96. doi: 10.18632/aging.204646 (PMC10120894; doi:10.18632/aging.204646)
Supplement: Supplementary Figures [file aging-15-204646-s001.pdf]

SUPPLEMENTARY FIGURES

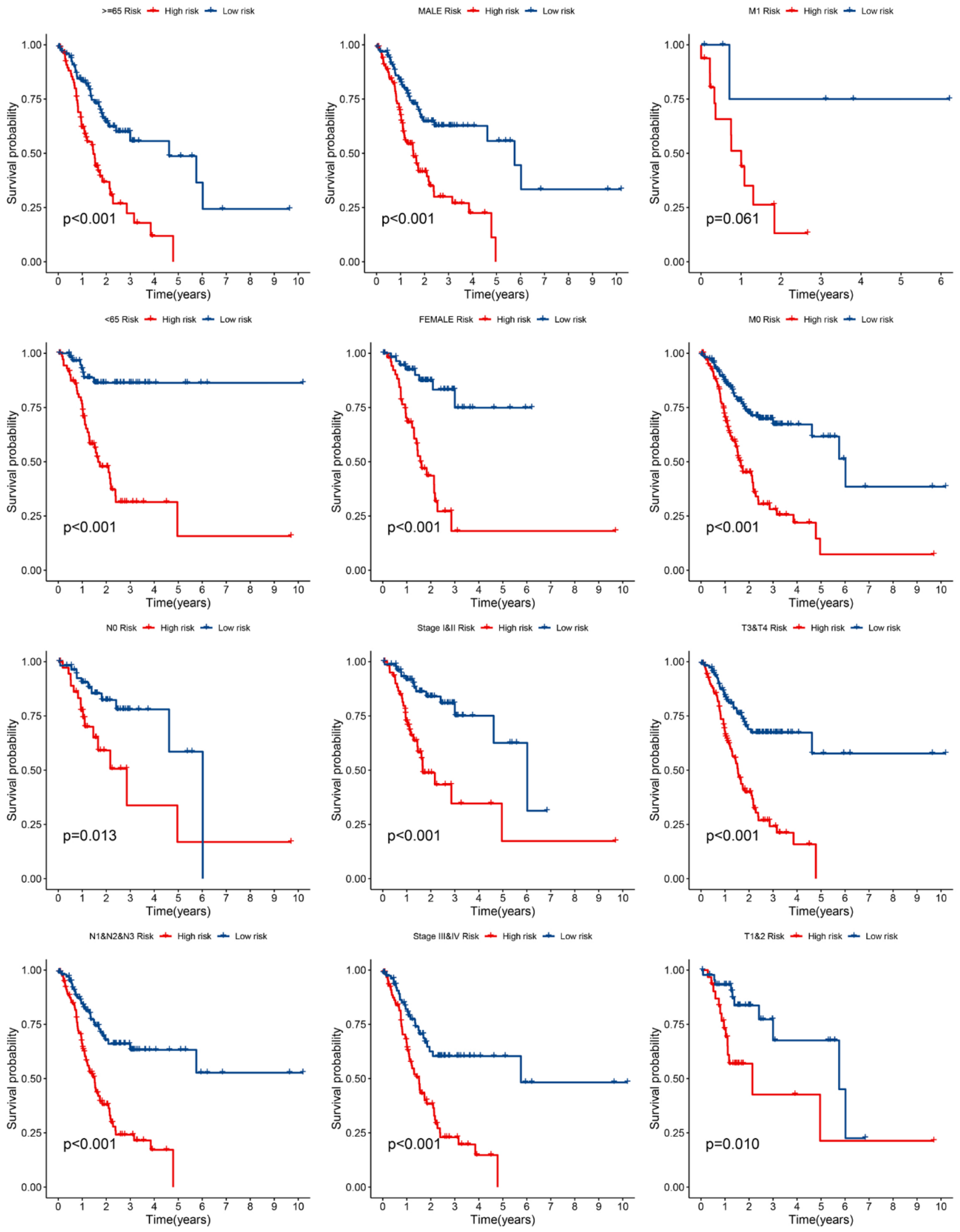

Supplementary Figure 1. Kaplan-Meier curves of OS differences stratified by age, gender, M stage, N stage, T stage, and pathological stage between the high-risk groups and low-risk groups.

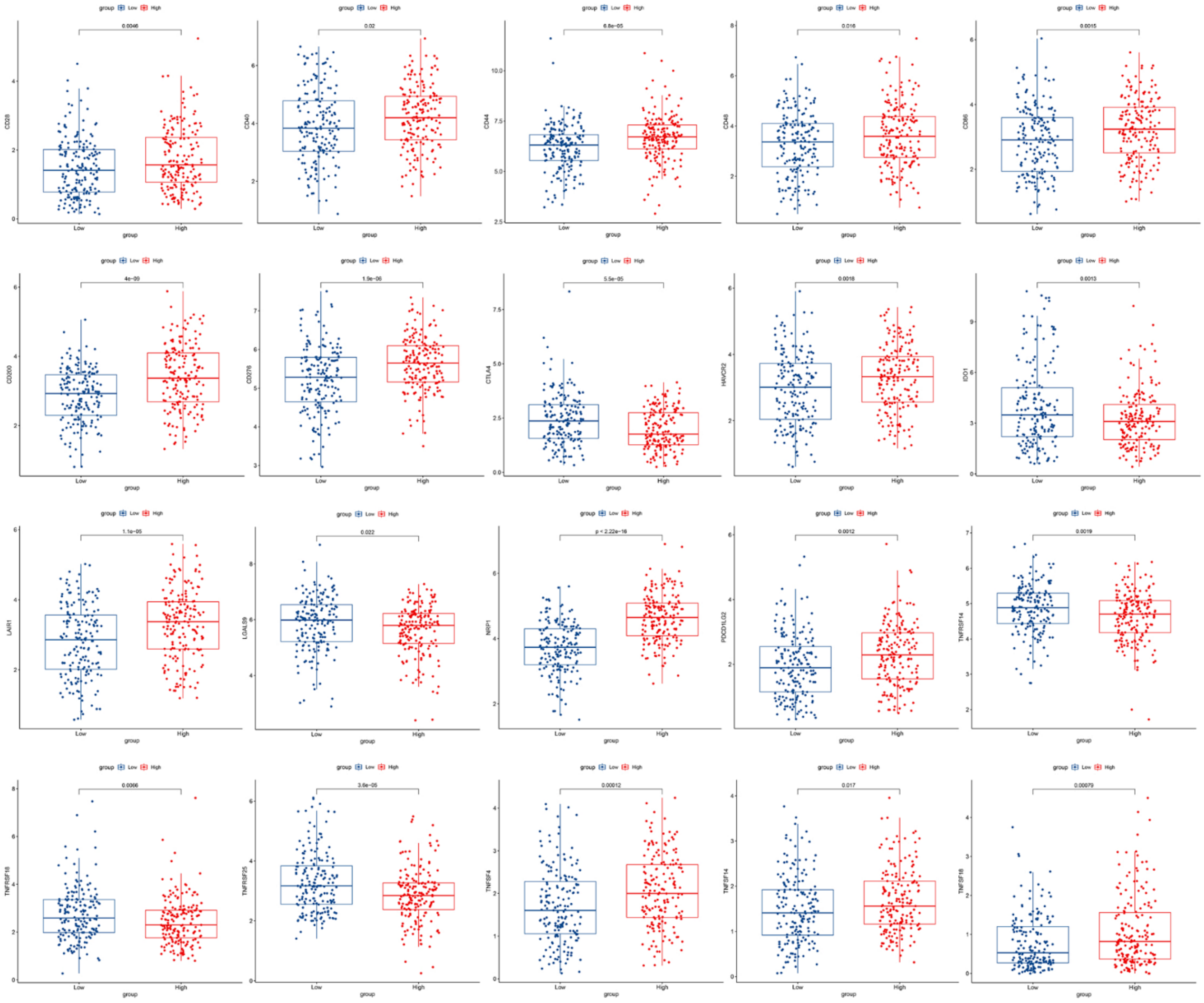

Supplementary Figure 2. The expression of immune checkpoints between high-risk groups and low-risk groups.

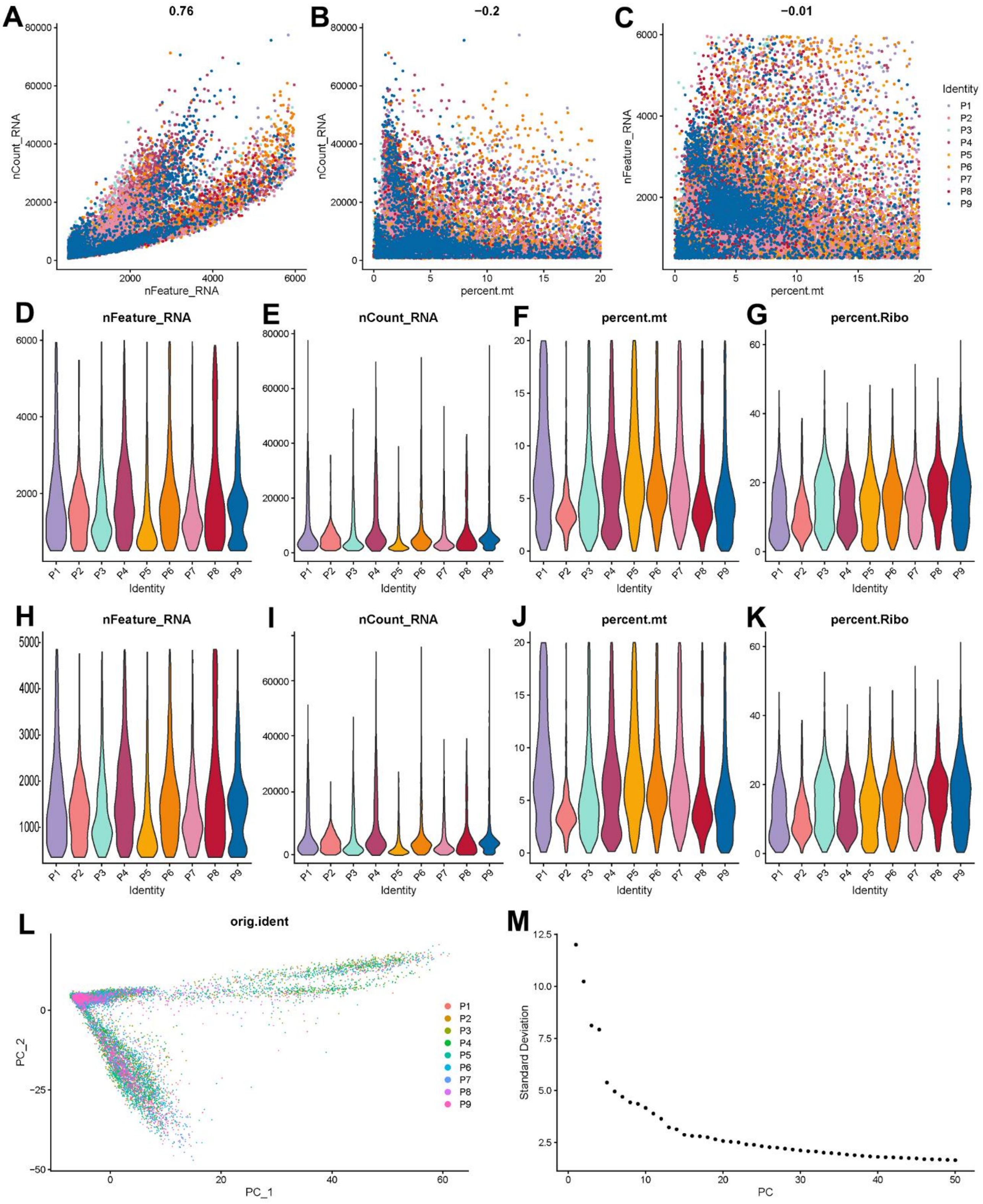

Supplementary Figure 3. Quality control of GC single cells. (**A**–**C**) Correlation analysis between nFeature and nCount, mitochondrial gene ratio and nCount, mitochondrial gene ratio and nFeature among different samples. (**D**–**G**) Violin plots of nFeature, nCount, mitochondrial gene ratio and hemoglobin gene ratio between different samples before quality control screening. (**H**–**K**) Violin plots of nFeature, nCount, mitochondrial gene ratio and hemoglobin gene ratio between different samples after quality control screening. (**L**) Cell PCA dimensionality reduction analysis of different samples. (**M**) The reference map of principal components (PCs) selection.
